# Morphometric Characterization of Human Coronary Veins and Subvenous Epicardial Adipose Tissue—Implications for Cardiac Resynchronization Therapy Leads

**DOI:** 10.3389/fcvm.2020.611160

**Published:** 2020-12-08

**Authors:** Jonas Keiler, Felix G. Meinel, Jasmin Ortak, Marc-André Weber, Andreas Wree, Felix Streckenbach

**Affiliations:** ^1^Department of Anatomy, Rostock University Medical Center, Rostock, Germany; ^2^Institute of Diagnostic and Interventional Radiology, Pediatric Radiology and Neuroradiology, Rostock University Medical Center, Rostock, Germany; ^3^Rhythmology and Clinical Electrophysiology, Divisions of Cardiology, Rostock University Medical Center, Rostock, Germany; ^4^Center for Transdisciplinary Neurosciences Rostock (CTNR), Rostock University Medical Center, Rostock, Germany

**Keywords:** cardiac anatomy, cardiac veins, epicardial fat, left ventricular pacing, human cardiac histology

## Abstract

Subvenous epicardial fat tissue (SEAT), which acts as an electrical insulation, and the venous diameter (VD) both constitute histomorphological challenges for optimal application and lead design in cardiac synchronization therapy (CRT). In this study, we characterized the morphology of human coronary veins to improve the technical design of future CRT systems and to optimize the application of CRT leads. We retrospectively analyzed data from cardiac computed tomography (CT) of 53 patients and did studies of 14 human hearts using the postmortem freeze section technique and micro CT. Morphometric parameters (tributary distances, offspring angles, luminal VD, and SEAT thickness) were assessed. The left posterior ventricular vein (VVSP) had a mean proximal VD of 4.0 ± 1.4 mm, the left marginal vein (VMS) of 3.2 ± 1.5 mm and the anterior interventricular vein (VIA) of 3.9 ± 1.3 mm. More distally (5 cm), VDs decreased to 2.4 ± 0.6 mm, 2.3 ± 0.7 mm, and 2.4 ± 0.6 mm, respectively. In their proximal portions (15 mm), veins possessed mean SEAT thicknesses of 3.2 ± 2.4 (VVSP), 3.4 ± 2.4 mm (VMS), and 4.2 ± 2.8 mm (VIA), respectively. More distally (20–70 mm), mean SEAT thicknesses decreased to alternating low levels of 1.3 ± 1.1 mm (VVSP), 1.7 ± 1.1 mm (VMS), and 4.3 ± 2.6 mm (VIA), respectively. In contrast to the VD, SEAT thicknesses alternated along the further distal vein course and did not display a continuous decrease. Besides the CRT responsiveness of different areas of the LV myocardium, SEAT is a relevant electrophysiological factor in CRT, potentially interfering with sensing and pacing. A sufficient VD is crucial for successful CRT lead placement. Measurements revealed a trend toward greater SEAT thickness for the VIA compared to VVSP and VMS, suggesting a superior signal-to-noise-ratio in VVSP and VMS.

## Introduction

In contrast to coronary arteries, which are the main focus of public attention, investigation of and literature on the coronary venous system (CVS) are scarce. Despite this, the clinical importance of the CVS is undisputed (1, 2); this is reflected by several electrophysiological methods employed using the CVS, such as radiofrequency catheter ablation, percutaneous mitral annuloplasty or cardiac resynchronization therapy (CRT) (3, 4). Such interventional procedures rely on detailed knowledge of the coronary venous anatomy to result in satisfactory success rates (5).

As a current standard in CRT, a transvenous quadripolar lead is implanted epicardially in the CVS of the left ventricle (LV) to synchronize the function of both ventricles with a pacemaker (6). CRT is indicated in patients with heart failures who were suffering from a low ejection fraction (LVEF ≤ 35%) or prolonged QRS duration (≥150 ms) (7) and therefore have a higher risk of sudden cardiac death (8).

Successful CRT requires appropriate lead placement, but the individual anatomy of the cardiac veins is often a limiting factor (9). In some cases, CRT nonresponse occurs because of suboptimal lead positioning, e.g., in a myocardial scar with a persistent irregular heart rhythm (10). Subvenous epicardial fat tissue (SEAT), which acts as an electrical insulation, and the venous diameter (VD) both constitute histomorphological challenges for optimal left ventricular lead application and lead design; this has been shown to hold true in studies of the porcine heart (11, 12).

There are a large number of studies dealing with epicardial fat tissue and the associated effects on coronary arteries such as increased risk of ischemic heart disease, cardiac hypertrophy and atrial fibrillation (13). However, morphometric data regarding coronary veins in the human heart is limited, data regarding SEAT is extremely scarce (14).

In clinical routines, various examination modalities are used for the morphological evaluation of the CVS. Computed tomography (CT) is an established technique for preoperative mapping of the cardiac veins to assess the vascular structure (15).

However, even the most modern CT systems are limited in their spatial resolution (16). Therefore, a detailed assessment of small structures such as the SEAT requires an *in vitro* assessment using, for example, micro computed tomography (mCT) (or X-ray microscopy) or histological approaches (17).

By examining the CVS and the adjacent tissue structures with different modalities, a more detailed anatomical comprehension should be possible. Therefore, the aim of our study was to assess morphometric parameters of the CVS and its adjacent tissues to improve the technical design of future CRT systems and to optimize the application of related pacemaker leads. The different methods in our multimodal approach are discussed and evaluated.

## Materials and Methods

This study was approved by the local ethics committee (A 2019-0014) with waiver of informed consent. This study was conducted in accordance with the Declaration of Helsinki on the ethical principles for medical research involving human subjects from October 2013.

### Computed Tomography (CT)

We used consecutive cardiac CT scans performed in preparation of an upcoming transcatheter aortic valve implantation (TAVI) at our center between July 2018 and April 2019, employing a 64-detector CT system (Aquilion 64, Canon Medical Systems, Germany). Patients were examined in the supine position and with deep inspiration breath-hold. No premedication was given. The examinations were performed with retrospective ECG gating. Tube voltage was 120 kV. A bolus of 90 mL of iodinated contrast (Imeron® 400MCT, Bracco Imaging Deutschland, Germany) was injected at a flow rate of 4 mL/s, followed by a saline chaser. The scan was started by bolus triggering in the ascending aorta. Transverse images in the end-systolic phase were reconstructed with a slice thickness of 0.5 mm.

Of 63 datasets of consecutive patients, 10 were discarded due to poor image quality or poor opacification of the coronary veins. The remaining 53 datasets were used for the segmentation-based reconstruction of the coronary vein tree and relative tributary positions. Patients had a mean age of 74.4 ± 10.3 years and weighed 80.7 ± 13.9 kg on average. Proportion of women was 39.7%. All subjects were Caucasian.

Detailed measurements of the VD and SEAT thickness were done for 10 consecutive exemplary datasets with 30% females and a mean age of 71.7 ± 10.1 years (see section Reconstruction and measurement of CT- and mCT-datasets).

### Dissection and Preparation

For the histological examination by frozen section technique (FST), four hearts were taken from suitable body donors (time of death <36 h before removal). Mean age was 87.8 ± 8.4 years, one was male (details see Supplementary Table 1). After a short rinse (3–5 min) in cold neutral phosphate buffered saline (PBS), the hearts were fixed in 3.7% neutral buffered paraformaldehyde (PFA) and stored at 4°C. Shortly before preparation of the coronary veins, the hearts were transferred to 0.1 M PBS (pH 7.4) and continuously stored at 4°C.

For the examination by means of mCT, 10 previously fixed hearts from body donors (50% male) were prepared. During their lifetimes the body donors (mean age 82.6 ± 10.5 years; details see Supplementary Table 1) had been treated with a pacemaker or an implanted defibrillator or had suffered from coronary heart disease (CHD). The hearts were fixed immediately after removal by immersion in 3.7% PFA or aqueous formaldehyde solution (FA). For further examinations, the hearts were transferred to and stored in 70% ethanol.

Body donors were different individuals than the patients who were CT-scanned.

**Figure 1:**
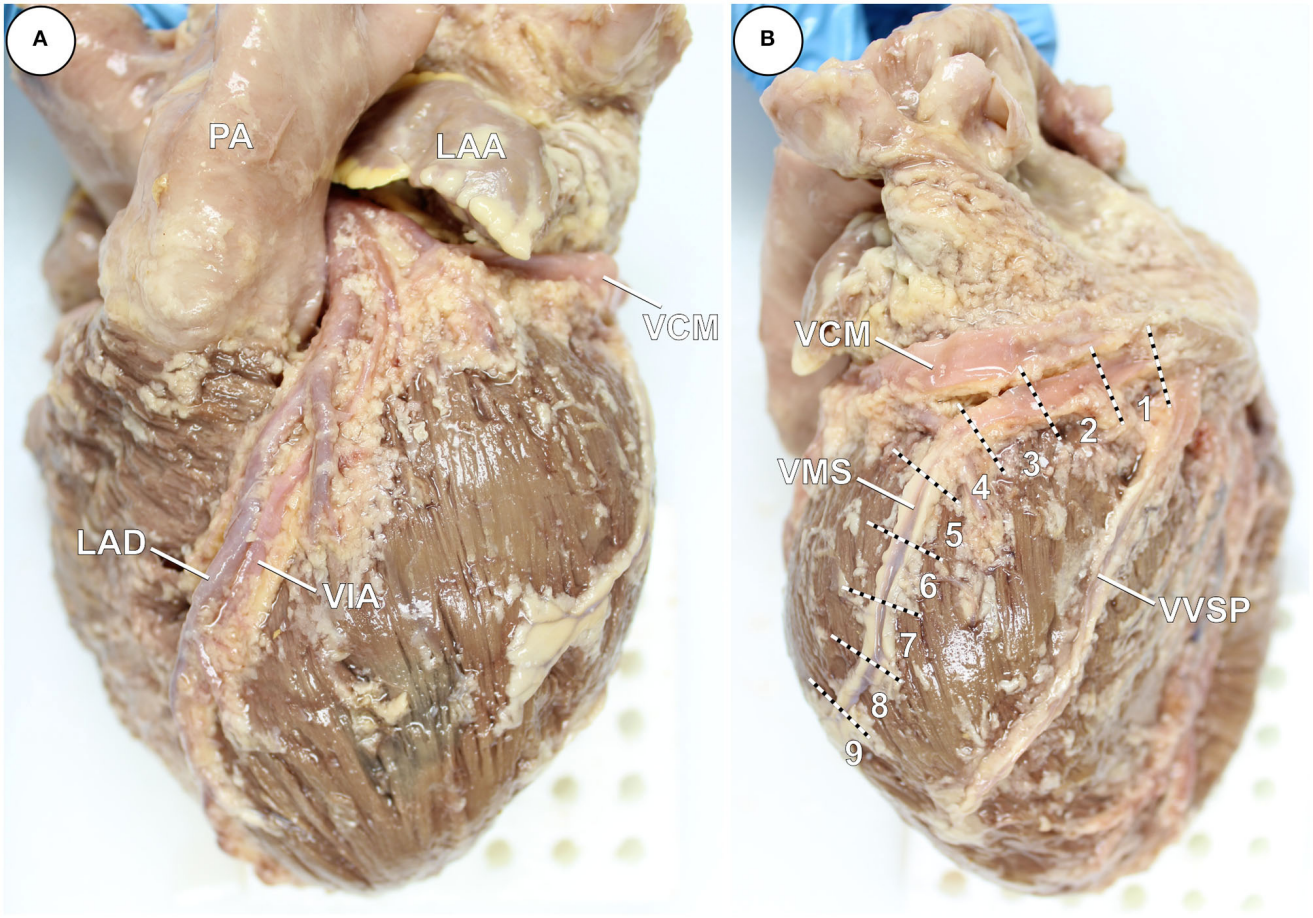
**(A,B)** PFA-fixed heart from a body donor with exposed coronary veins after preparation and removed epicardial adipose tissue. **(A)** Left anterolateral view. **(B)** Right posterolateral view. Specific segments (1–9) of the left marginal vein (VMS) are indicated by dashed lines. LAA, left atrial appendage; LAD, left anterior descending artery; PA, pulmonary artery; VCM, great cardiac vein; VIA, anterior interventricular vein; VVSP, left posterior ventricular vein.

To identify the individual coronary veins in the epicardial adipose tissue, the coronary vessels were superficially dissected (Figure 1) followed by photographic documentation. The morphology and dimensions of the Thebesian valve, shielding the ostium of the coronary valve, was assessed.

The parameters height, width, and depth of the ventricles were recorded using calipers to assess the respective heart size (mean of LV height and widest lateral and AP-axis). The common clinical parameter of the distance from the apex to the base of the heart was not examined because of the mostly collapsed atria.

Subsequently, the respective main stem of the coronary veins was dissected with preservation of the upper myocardial layer tissue, subvenous adipose tissue and coronary vein wall; these were documented photographically.

### Frozen-Section Technique (FST)

For histomorphological imaging based on the frozen-section technique (FST), the fixed tissue segments (*n* = 19) stored in chilled PBS were crosscut into sub-segments (*n* = 154) of 1 cm length each along the vascular axis and cryoprotected in 20% saccharose. Subsequently, sub-segments were quick-frozen in isopentane at −80°C for 5 min and then stored at −20°C. The frozen segments were then cut at −17°C and a slice thickness of 50 μm in a cryostat (CM3050 S, Leica Biosystems, Germany). For each segment, several stages (2–6) with > 1 mm distance between sequential stages were cut, mounted and dried. Slices (*n* = 1,423) were digitized with a Leica DM6 bright field microscope at 10× magnification and using the tiles-merge-application within the device software to assess the whole slice.

### Micro Computed Tomography (mCT)

The tissue structure of fixed coronary veins stored in ethanol was examined using mCT. The images were taken with a Phoenix Nanotom-180 (GE sensing and inspection technologies, USA) with 50 kV and 300 μA at a resulting voxel resolution of 23 μm. The ^*^.tiff image stacks (exported with VGStudio Max, Volume Graphics, Germany) were then further analyzed (see section Measurement of cryosections).

### Measurements

#### Reconstruction and Measurement of CT and mCT Datasets

**Figure 2:**
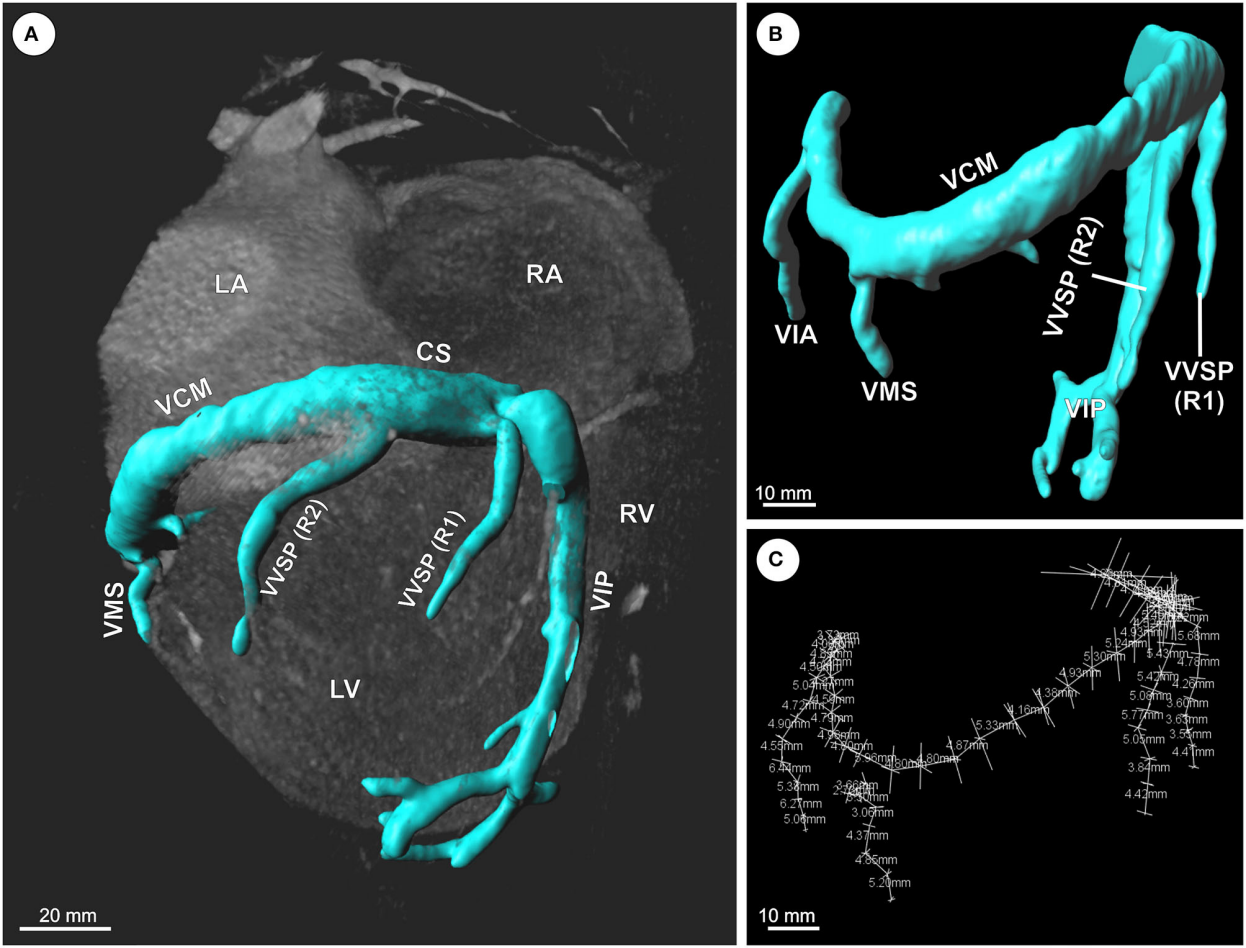
**(A–C)** Exemplary 3D reconstruction and morphometric analysis of the coronary vein system (CVS) based on CT datasets. **(A)** Volume rendered CT image of the heart with overlaid surface model of the CVS. **(B)** Reconstructed surface model of the CVS with overlaid orthogonal measurements every 5 mm. **(C)** Measurement points and lines along the CVS. CS, coronary sinus; LA, left atrium; LV, left ventricle; RA, right atrium; R1/R2, 1st/2nd ramus; RV, right ventricle; VCM, great cardiac vein; VIA, anterior interventricular vein; VIP, posterior interventricular vein (=middle cardiac vein); VMS, left marginal vein; VVSP, left posterior ventricular vein.

Anonymized CT datasets were exported as Tiff images using MicroDICOM viewer (MicroDicom, Bulgaria). Anisotropic voxel size was at a minimum of 300 μm and a maximum of 782 μm edge length. Subsequently, Tiff images were imported into the 3D reconstruction software Imaris 8.4 (Bitplane, Switzerland). At first, manual segmentation of the coronary vein course, starting at the proximal ostium, was performed, followed by threshold-based generation of a vascular surface model (Figure 2). For 10 datasets, measurements of VD and SEAT were done along the course of the veins by setting orthogonal slices (“oblique slicer”) every 5 mm. For coronary sinus (CS) and great cardiac vein (*V. cardiac magna*; VCM), VD was measured proximal to the tributary of the posterior interventricular vein (*V. cardiac media* = *V. interventricularis posterior*; VIP). For each orthogonal slice, the vein diameter was averaged from the LR and the AP diameter. The thickness of the underlying subvenous fat layers was averaged from three measurements for each slice.

The distances of the main left ventricular tributaries of CS and VCM relative to the ostium of the CS were measured for 53 datasets. Corresponding to the body donor hearts, the parameters height, width and depth of the ventricles were measured in the CT data to assess the respective heart size.

**Figure 3:**
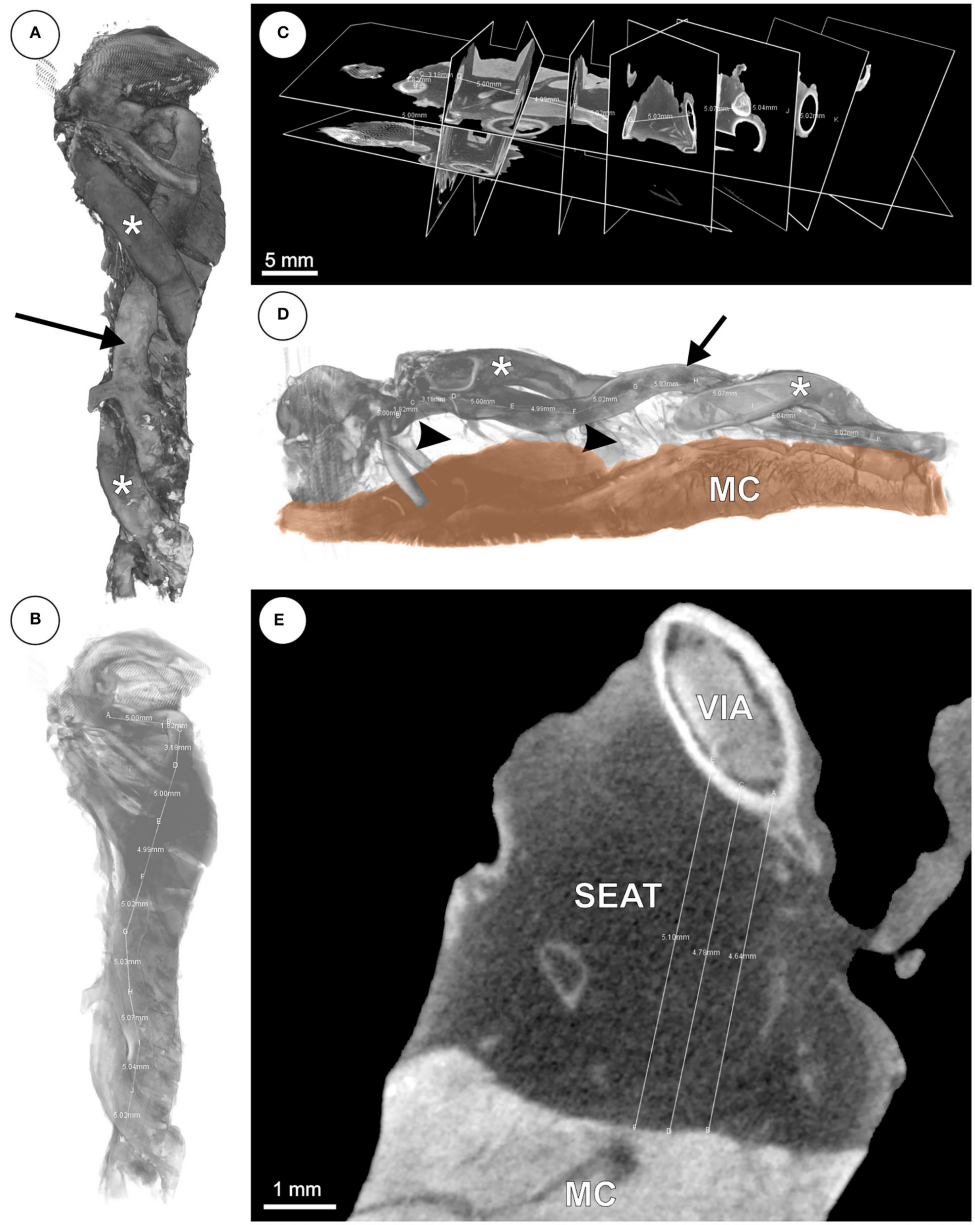
**(A–E)** mCT based 3D reconstruction of the anterior interventricular vein (VIA). **(A)** Volume rendering of the VIA (arrow) with adjacent branches of the left anterior descending artery (LAD; asterisks). **(B)** Volume from panel **(A)** with decreased opacity and distance measurement. **(C)** Orthogonal slices of the VIA for distance measurements every 5 mm. **(D)** Volume rendering of the VIA (arrow) with branches of the LAD (asterisks), underlying adipose tissue (arrow heads) and ventricular myocardium (MC). **(E)** Virtual slice with subvenous epicardial adipose tissue (SEAT) and distance measurements between VIA and MC.

For the examination of the mCT datasets (Figure 3), exported Tiff stacks were loaded into the Imaris software, followed by measurements of VD and SEAT along the course of the veins by setting orthogonal slices (“oblique slicer”) every 5 mm. Since vein walls were collapsed in post-mortem tissue, VD was calculated from the inner vascular perimeter for each orthogonal slice, assuming an ideal circular shape in cross-section.

#### Measurement of Cryosections

**Figure 4:**
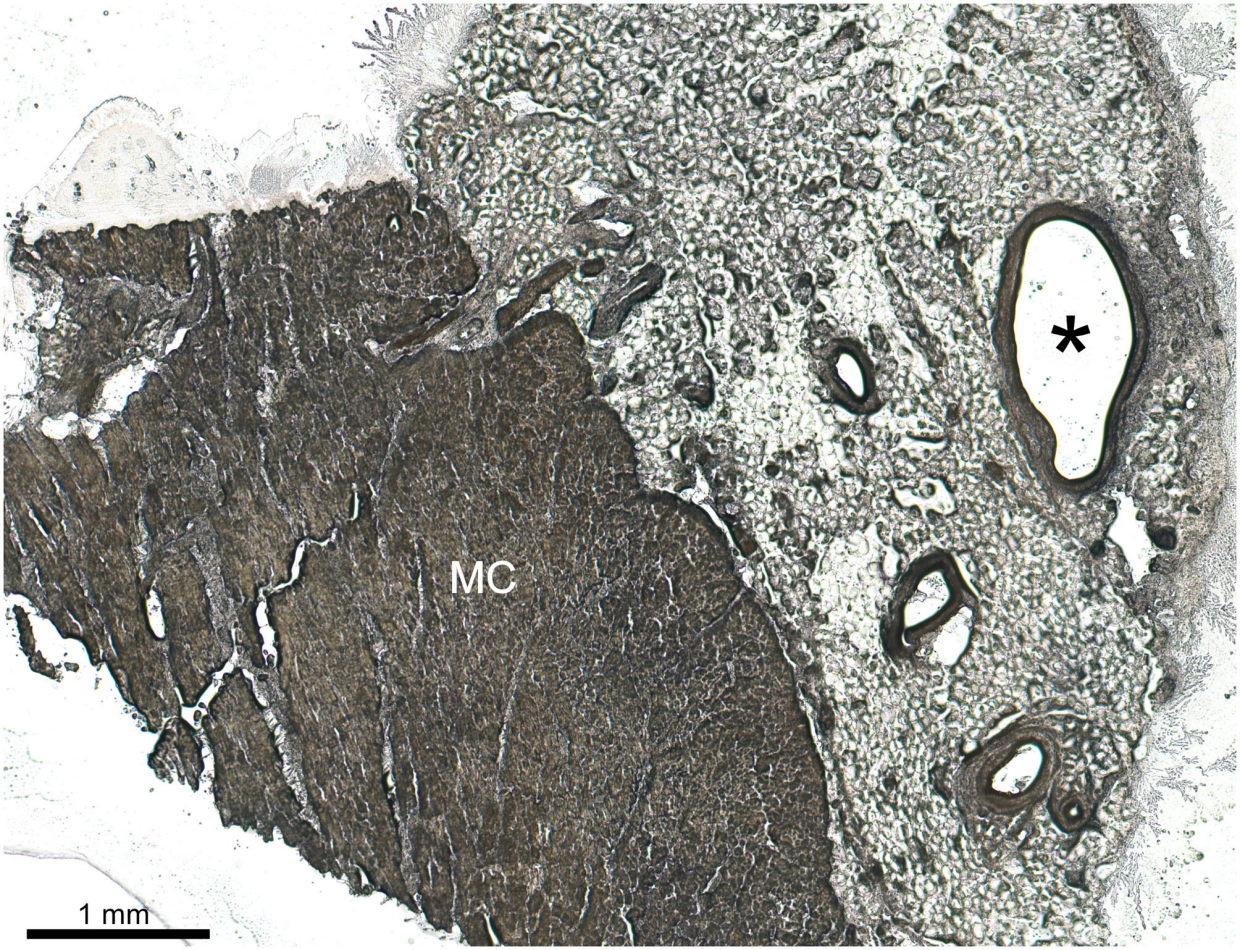
Cryosection (50 μm thick) of left posterior ventricular vein (*V. ventriculi sinistri posterior*; asterisk) embedded in epicardial adipose tissue and underlying left ventricular myocardium (MC).

The digitized frozen sections (Figure 4) were analyzed with the measuring tool “Analyze” of the ImageJ/Fiji software. The inner circumference as well as the distance between the inner vein wall and the interface between left ventricular myocardium and SEAT were measured. Only orthogonal sections were used to measure the inner circumference. Longitudinal or oblique sections were excluded from the calculation of the inner diameter. The SEAT thickness was calculated by the mean value of three measurements in an area with the shortest distance between venous lumen and myocardium. The VD was calculated according to the inner circumference assuming an ideal circular profile. The measurements of each section stage were averaged and then evaluated using the digital images. The values for each vein segment with a respective length of ~1 cm were averaged from the prior averaged cutting-stage values to smooth possible outliers due to inflows in the section plane or oblique sections.

### Statistics

Significance tests were calculated using GraphPad Prism 8.0.2. Normality distribution was checked using the Shapiro–Wilk test. Mean value comparison was done using the Wilcoxon test for non-parametric data or the ratio *t*-test for parametric data. Significant differences were accepted for *p* < 0.05.

## Results

### Tributary Topography

**Figure 5:**
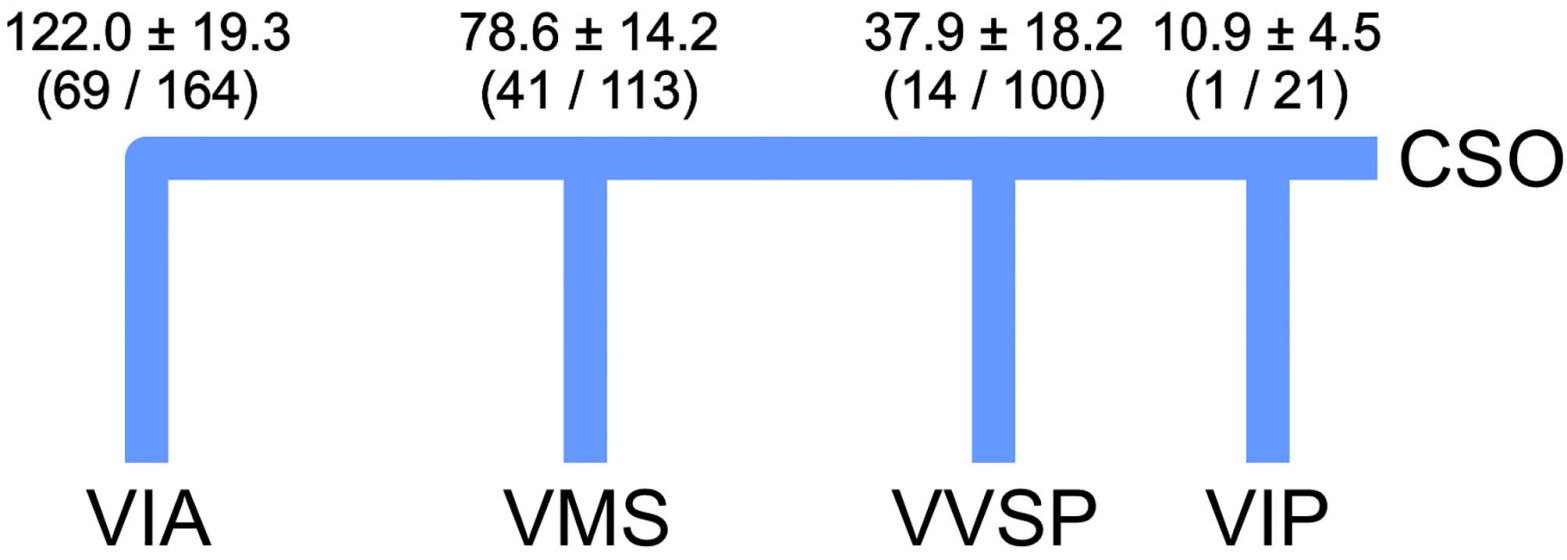
Mean position ± standard deviation of the venous tributaries as relative distances to the ostium of the coronary sinus (CSO) inspected by CT (*n* = 53). Values include double tributaries for VVSP (6 × 2) and VMS (1 × 2). Minimum and maximum values in brackets. Values are given in mm. VIA, *V. ventricularis anterior*; VIP, *V. interventricularis posterior*; VMS, *V. marginalis sinistra*; VVSP, *V. ventriculi sinistri posterior*.

The distances of the main left ventricular tributaries of CS and VCM relative to the ostium of the CS were measured for 53 CT datasets (Figure 5). The posterior interventricular vein (*V. interventricularis posterior*, VIP) entered the CS between 1 and 21 mm distal to the ostium of the CS (CSO). The posterior vein of the right ventricle (*V. ventriculi sinistri posterior*, VVSP) reached the CS/VCM between 14 and 100 mm distal to the CSO. The left marginal vein (*V. marginalis sinistra*, VMS) entered the CS/VCM between 41 and 113 mm, followed by the branch of the anterior interventricular vein (*V. interventricularis anterior*, VIA) between 69 and 164 mm. For none of the tributaries did branch distance to the CS ostium correlate with heart size (*R*^2^ ≤ 0.18). Double tributaries were detected in the six cases for the VVSP and in one case for the VMS. In three cases no VVSP, in five cases no VMS, and in one case no VIA could be detected in the CT-based volumes—mainly due to insufficient structural resolution in the area concerned.

Values for the oblique vein of the left ventricle (*V. obliqua atrii sinistri*) and the right ventricular small coronary vein (*V. cardiaca parva*) were not assessed.

The tributary angles θ (between parent and branching daughter vessel) averaged 114.3° for all distal main tributaries (VVSP, VMS, and VIA) of 10 exemplary hearts, with a mean 103 ± 29° for VVSP, 110 ± 23° for VMS and 130 ± 29° for VIA. One VCM was consistently sloped, making angle measurement not feasible, another was strongly meandering into the VIA.

### VD and SEAT Thickness

For the morphometric assessment of VD and SEAT thickness, 10 reconstructed coronary venous systems based on clinical CT volumes were analyzed, and tissue samples from 14 hearts derived *postmortem* from body donors were examined by means of FST and mCT. The VD and SEAT values of the different individuals were compared centimeter for centimeter by means of their relative position within the coronary vein tree (Figure 2).

#### Venous Diameter (VD) (Figure 6)

At its proximal portion, the CS/VCM had the greatest average VD (13.8 ± 5.2 mm) ranging from 5.1 to 25.0 mm. The VD decreased to a mean of 7.5 ± 1.4 mm in the middle portion (5 cm position) and further decreased to 5.3 ± 1.4 mm more distally (10 cm position). The VVSP possessed a mean VD of 4.0 ± 1.4 mm in the proximal portion, ranging from 1.6 to 6.6 mm. More distally (5 cm position), the VD decreased to 2.4 ± 0.6 mm on average. The VD of the VMS started with 3.2 ± 1.5 mm in the proximal portion, decreasing to 2.3 ± 0.7 mm more distally (5 cm position). The distal-most tributary, the VIA, had a mean VD of 3.9 ± 1.3 mm on average, ranging from 1.4 to 6.5 mm. More distally (5 cm position), the VD decreased to 2.4 ± 0.6 mm. For none of the tributaries did VD correlate with heart size (*R*^2^ ≤ 0.18). For a detailed list of the assessed VDs see Supplementary Table 2. Scatter plots with individual data points can be consulted in Supplementary Figure 1.

**Figure 6:**
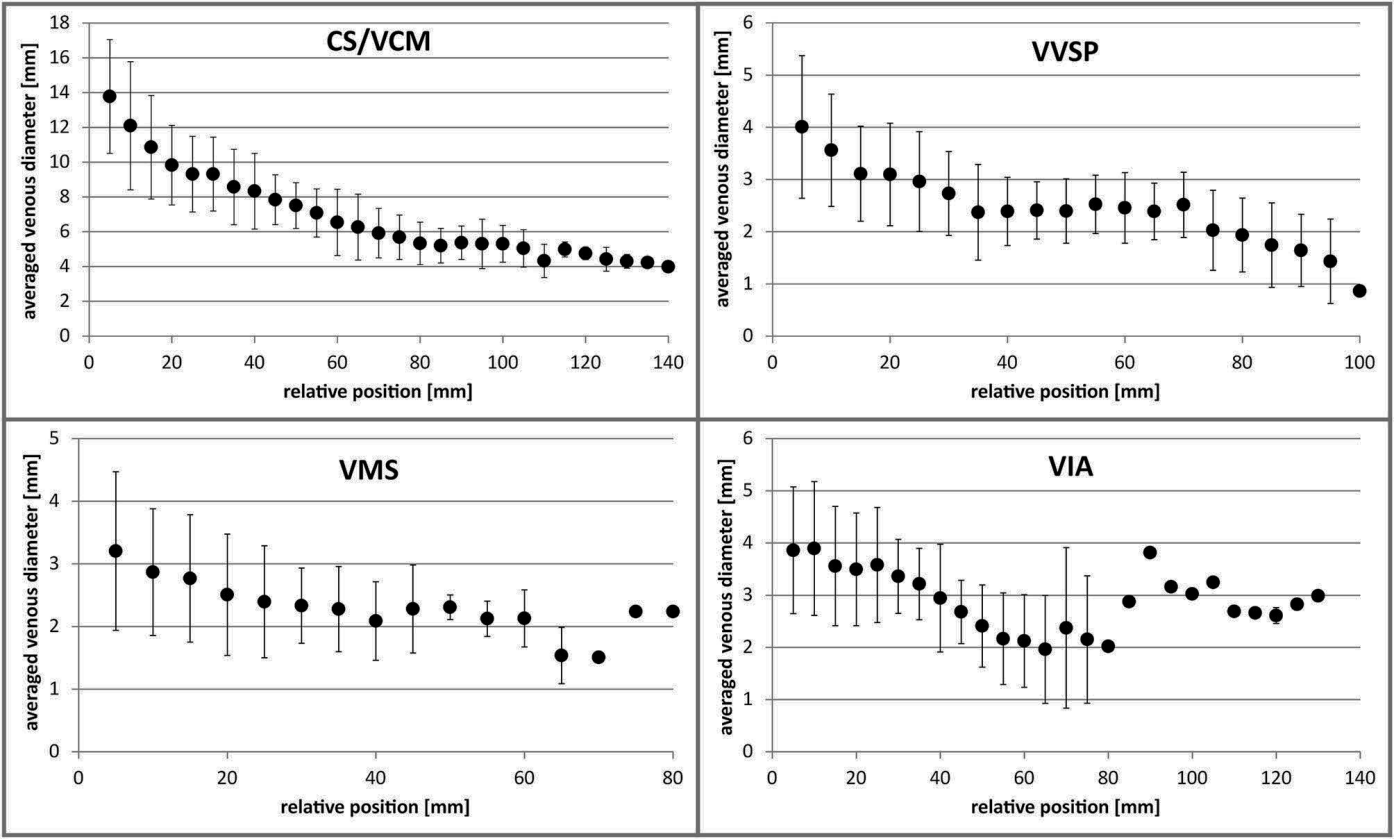
Averaged venous diameters along the distance of the different coronary veins. Error bars indicate standard deviation. Distal values without error bars are single measurements. CS, coronary sinus; VCM, great cardiac vein; VIA, anterior interventricular vein; VMS, left marginal vein; VVSP, left posterior ventricular vein.

#### Subvenous Epicardial Adipose Tissue (SEAT) Thickness (Figure 7)

The mean SEAT thickness of the main CS/VCM tributaries was greatest in the proximal portion of the veins. In their proximal 15 mm, the veins had a SEAT layer with a mean thickness of 3.2 ± 2.4 mm for the VVSP (*n* = 16; min: 0.5 mm, max: 11.1 mm), 3.4 ± 2.4 mm for the VMS (*n* = 15; min: 0.4 mm, max: 7.9 mm), and 4.2 ± 2.8 mm for the VIA (*n* = 17; min: 0.3 mm, max: 12.4 mm). More distally (20–70 mm), the SEAT thickness decreased to a mean level of 1.3 ± 1.1 mm for the VVSP (*n* = 15) and 1.7 ± 1.1 mm for the VMS (*n* = 9). For the VIA (*n* = 16), mean seat thickness remained high with a mean level of 4.3 ± 2.6 mm; this decreased only slightly more distally. In a direct comparison of the distal portions, the VIA possessed SEAT at least 1.25 times thicker than the VVSP in 85.7% (*n* = 12/14; *p* = 0.0004 for paired differences) and the VMS in 88.9% (*n* = 8/9; *p* = 0.001 for paired differences) of the individuals.

**Figure 7:**
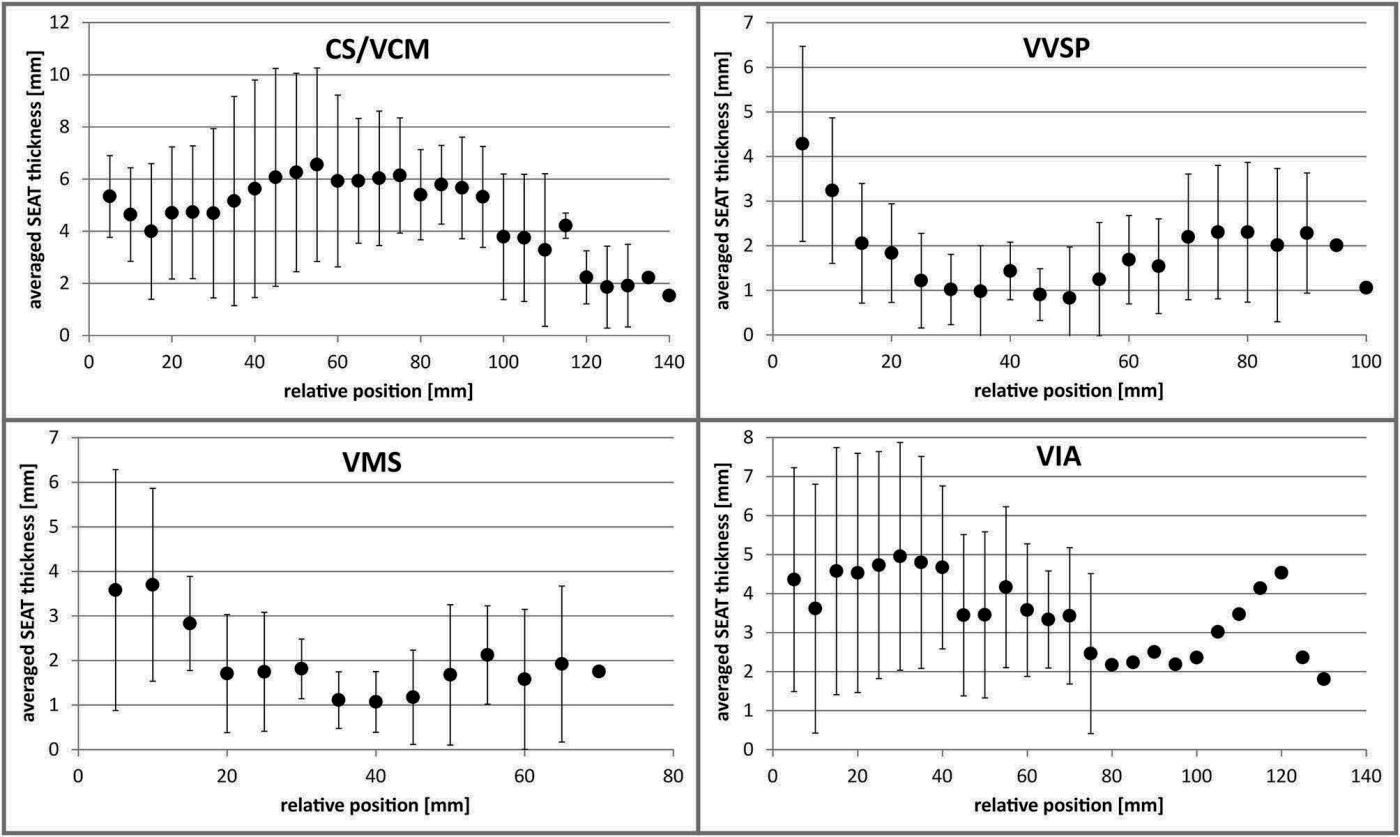
Averaged SEAT thickness along the distance of the different coronary veins. Error bars indicate standard deviation. Distal values without error bars are single measurements. CS, coronary sinus; VCM, great cardiac vein; VIA, anterior interventricular vein; VMS, left marginal vein; VVSP, left posterior ventricular vein.

In contrast to the VD, SEAT thicknesses alternated along the further distal vein course and did not decrease continuously. For a detailed list of the assessed SEAT thicknesses see Supplementary Table 3. Scatter plots with individual data points can be consulted in Supplementary Figure 2.

VD and SEAT values for the middle cardiac vein (*V. interventricularis posterior*), oblique vein of the left ventricle (*V. obliqua atrii sinistri*), and the right ventricular small coronary vein (*V. cardiaca parva*) were not assessed.

#### Thebesian Valve Morphology

The CS guarding Thebesian valves of the body donor hearts examined were largely crescent-shaped. The valve height varied between individuals and averaged 4.3 ± 2.1 mm (min: 1.5 mm, max: 8.0 mm). Valve size did not correlate with ostium size (*R*^2^ = 0.0014).

## Discussion

The CVS is a rather neglected anatomical structure, at least in the public perception, given the major role of the coronary arteries in coronary heart disease. Nonetheless, the clinical significance of the CVS is not to be underestimated. In contrast to the coronary arteries, the CVS is rather part of the problem solving than of the problem in most clinical cases. Besides catheter ablation of faulty conductive tissue (18) and mitral annuloplasty (19, 20), the implantation of leads for cardiac resynchronization therapy (CRT) is one of the most relevant percutaneous interventions using the CVS. In German hospitals alone, more than 12,000 CRT systems for left ventricular arrhythmia therapy are implanted each year; this represents 12% of all annual cardiac implantable electronic devices and the number is rising (21, 22).

### Significance of the Coronary Vein Morphology in CRT

Several aspects in coronary vein morphology are critical for successful lead placement in CRT. The coronary VD limits the maximal diameter of any CRT lead, although dilatation is feasible to some degree (23, 24). On the downside, bigger lead diameters affect venous hemodynamics and hinder blood drainage, thereby increasing the risk of thromboembolic events (25). Moreover, intense contact between the lead and the endothelium promotes thrombo-fibrotic lead encapsulations (26, 27) which can cause complications in the case of the indicated lead explantation (21, 28–30).

Present CRT leads have distal diameters ranging from 4.0 to 5.4 Fr (1.2–1.8 mm) which is rather thin in comparison to right ventricular pacing or defibrillation leads with up to 8.6 Fr (2.8 mm). It has been shown that the VD of the CS in systole is usually greater than in diastole, which can be ascribed to the phasic pattern of the coronary venous flow during the cardiac cycle (3).

As previously shown, close contact between lead electrodes and vein wall lowers the pacing threshold (12). This indicates that, although resulting in venous occlusion, a broader lead fully filling the vein lumen would potentially increase the probability of improved, i.e., lower, thresholds in the preferred pacing region. Via various anastomotic connections, accessory Thebesian veins could compensate the occluded venous drain into the right ventricle (RV) (31).

In a previous study, the CS ostium diameter correlated with heart size (32). We did, however, not observe a correlation between heart size and coronary VD.

Another critical aspect is the topography and prevalence of valves. Although physiologically of rather minor importance (33) and variably present (34–37), valves in the coronary veins are known to interfere with successful lead placement in the retrograde direction (36, 38). They are present either as ostial or parietal valves, most prominently the Thebesian valve of the CS ostium and the Vieussens valve between CS and VCM (39). Although we have occasionally recognized cuts of valve leaflets in our cryo-sections, we did not further quantify their presence due to potential error-proneness related to section clippings.

Besides valves, the venous route can pose another anatomical barrier for the application of the lead. Serpentinous or meandering courses of the vessel, which we have occasionally observed, may hinder the placement of the lead (40). Several data on branching angles of the emanating tributaries exist (41–44); these are in the scale of our measurements. Regarding the angle at which the tributaries emanate from the VCM/CS, an obtuse angle is most advantageous for the application of CRT leads.

Consistent with our findings, previous works have shown that the presence of the coronary veins is variable while the great cardiac and posterior interventricular (=middle cardiac) vein are constantly present (3, 41, 42, 45, 46). This limits the accessibility of the anterolateral, lateral and posterolateral LV regions. The averaged branch positions in our examination are largely in line with those in previous studies (41, 47, 48).

### SEAT and CRT Lead Position

Our findings on human hearts are corroborated by previous studies on human and porcine hearts (11, 14): coronary veins are underlaid by epicardial adipose tissue of varying thickness (Table 1). Generally, fat is an effective electrical insulator (51, 52). Due to the insulation properties of SEAT, which affects the sensing and pacing capture threshold (PCT) in CRT, SEAT thickness is suggested to be a relevant anatomical factor for CRT efficiency (11). In an histology-based simulation model, it was shown that SEAT lowers electrical field energy of transvenous electrodes, which in turn increases thresholds for myocardial activation (12). Basically, epicardial fat mass is correlated with body adiposity (53). Due to the sparse data situation, however, a link between SEAT thickness and obesity is unknown. Significantly higher pacing thresholds or sensing problems in obese patients have, to our knowledge, hitherto not been reported.

**Table 1:** Morphometric studies on human coronary vein morphology.

**Study**	***n***	**cohort type**	**Age (y)**	**Males (%)**	**Methods**	**VD CS (mean/range)**	**VD VVSP (mean/range)**	**SEAT VVSP (mean/range)**	**VD VMS (mean/range)**	**SEAT VMS (mean/range)**	**VD VIA (mean/range)**	**SEAT VIA (mean/range)**
1	24	*iv, pm*	78.9 ± 11.6	54.2	CT, mCT, FST	13.8 ± 5.2/ 5.1 – 24^*^	4.0 ± 1.4/ 1.6 – 6.6^*^	4.2 ± 2.7/ 1.0 – 11.1^*^	3.2 ± 1.5/ 0.6 – 5.5^*^	3.6 ± 2.2/ 1.2 – 7.3^*^	3.9 ± 1.3/ 1.4 – 6.5^*^	4.4 ± 2.2/1.3 – 9.9^*^
2	30	*iv*	–	–	CT	–	3.8 ± 1.5^**^	–	4.1 ± 1.1^**^	–	5.1 ± 1.2	–
3	6	*pm*	70.5 ± 14.6	16.7	Histology	–	2.2 ± 1.0^*^	1.8 – 3.0^*^	2.0 ± 0.6^*^	1.4 ± 1.1^*^	3.0 ± 0.6^*^	2.9 ± 1.2^*^
4	50	*iv*	60 ± 15	84.0	CT	13.8 ± 3.7^**^	–	–	2.7 ± 1.1/ 1 – 5	–	–	–
5	52	*pm*	58 ± 14	32.3	Balloon catheter	9.47 ± 3.7/ 6 – 13^**^	–	–	–	–	–	–
6	40	*pm*	13 – 68	–	Caliper	8.43 ± 2.6/ 5 – 20^**^	–	–	–	–	–	–
7	54	*pm*	–	–	Stereoscopy, histology	9/ 5 – 22	–	–	–	–	–	–
8	150	*pm*	24 – 84	61.0	Caliper	9.6 ± 2.2/ 3 – 19^*^	–	–	–	–	–	–
9	14	*iv*	64 ± 7		MRI	9.8 ± 3.0	3.3 ± 0.8	–	2.9 ± 0.6	–	–	–
10	19	*pm*	46 (19–75)	57.9	Caliper	–	5/ 1 – 15	–	6/ 2 – 13	–	–	–
11	37	*pm*	–	–	Caliper	8.8 ± 1.7/ 6.0 – 12.0^*^	2.4 ± 1.1/ 1.0 – 5.5^*^	–	2.3 ± 0.8/ 0.8 – 4.5^*^	–	2.7 ± 0.7/ 0.9 – 4.4^*^	–
12	255	*iv*	48.7 ± 19.4	49.4	Venography	–	2.3 ± 1.6	–	2.9 ± 1.5	–	–	–
13	200	*pm*	48.7 ± 15.6	78.0	Caliper	–	1.3 ± 0.5/ 0.5 – 3.3^**^	–	0.9 ± 0.5/ 0.1 – 2.4^**^	–	–	–

*VD, vein diameter in mm; SEAT, subvenous epicardial adipose tissue thickness in mm; (1) present study, (2) Spencer et al. (41), (3) Anderson et al. (14), (4) Christiaens et al. (43), (5) Karaca et al. (32), (6) Marić et al. (45), (7) Maros et al. (33), (8) Hellerstein and Orbison (49), (9) Manzke et al. (47), (10) Hood (50), (11) Ortale et al. (46), (12) Uhm et al. (44), (13) Mazur et al. (48). Iv, in vivo; pm, post-mortem*.

* *proximal*,

** *ostial*.

Consistent with our findings, Anderson et al. (14) found the VIA to have a significantly thicker SEAT than VMS and VVSP. Given this, a superior signal-to-noise ratio in sensing and a lower pacing threshold in CRT can be assumed for VMS and VVSP. Interestingly, a majority of clinical studies suggest better outcomes, e.g., in terms of mortality or reverse LV remodeling, etc., when the CRT lead is placed in the VMS and VVSP [e.g., (54–56)]. Various prospective clinical trials have shown that with quadripolar leads, which represent the current standard of care in CRT, the PCTs for non-apical LV electrodes are found to have “the best myocardial contact” [e.g., (6, 57)]. This is barely surprising regarding the distal anchoring tines in those models, which probably distance the electrode from the tissue. The most crucial aspect for an optimal CRT, however, appears to be pacing of the myocardial area with maximum delay (latest area of activation) (58, 59); in most cases this is the lateral and posterolateral LV region (56), corresponding largely with the coverage of VMS and VVSP. The topographic correlation indicated between veins with lower SEAT thickness and the area of latest activation is presumably coincidental and non-causal. Given this, lead placement and electrode positioning according to certain SEAT conditions are presumably subordinate. Nonetheless, fine adjustment in electrode positioning within the delayed area could optimize threshold values and battery longevity to some extent.

### Leadless Pacing in CRT

Current limitations in CRT related to vein morphology and SEAT could be overcome with recent developments of leadless LV pacemaker systems (60–62). Leadless systems generally have the advantage of nullifying the risk of lead failure and thrombo-fibrotic lead encapsulations (TFLE) or lead infections by minimizing implant size and implant-tissue interface equally. In the right ventricle (RV), however, leadless systems bear the risk of an embolic event due to detachment of the implant site (63). Implant detachment in the LV would not result in less severe complications by migrating into the arterial pathway. While early battery depletion is still a challenging risk in leadless RV systems, recent leadless LV systems use external pulse generators with ultrasonic and subcutaneous batteries as a power source (60). Similar to conventional pacemakers, pocket problems, including device-related infections, are post-operative complications with leadless CRT systems (60, 64). The usual retrograde transaortic implantation of the leadless devices into the LV often necessitates general anesthesia (64). A transvenous, transseptal implantation is an alternative and would be less invasive (61).

Recently some authors questioned the necessity of leadless CRT systems (65). However, leadless CRT systems are beneficial for up to half of non-responders to conventional CRT (60, 64).

Although leadless biventricular pacing has been shown to be feasible and effective (61, 62), it is limited to patients without an implantable cardioverter defibrillator (ICD) since endocardial defibrillation has (currently) no leadless alternative. CRT-D patients, however, constitute ~70% of all CRT patients and still rely on a subcutaneous pulse generator and transvenous leads. Thus, a combination of a transvenous ICD with a leadless LV system still retains the risks of pocket infections and TFLE in the afferent veins, in addition to the above-mentioned risks for leadless LV devices. The feasibility of subcutaneous defibrillation (66) and leadless biventricular pacing has yet to be proven.

### Strengths and Limitations of the Study

One advantage of our study is the multimodal approach which allowed a comprehensive morphometric characterization of the coronary veins, although not statistically powered due to the limited sample size. Examination of the CT-based volumes provided profound topographic information, whereas resolution limited the small-scale reconstruction and measurement.

Although the CT scans were performed to assess the aortic and arterial morphology, retrograde venography (RVG) would have provided better visualization of the coronary veins. However, image quality in the datasets studied herein was sufficient, and the accessible sample size was bigger than for RVG. FST and mCT in turn allowed high resolution imaging but had their limitations in topographic integrity after dissection. Occasional non-orthogonal slicing in FST could have provided biased absolute measurement values in some cases.

The lengths of the measured vein portion varied between the different individuals, as the distal portion of the veins was not always identifiable during macroscopic preparation or in the reconstructed CT volume. In those cases the measurement was limited to the proximal section along the basal and mid-ventricular region of the heart which are, following previous studies, preferred pacing regions with better outcomes after CRT compared with pacing in the apical region (67).

The selection of CT data from TAVI candidates provides for a certain bias in our study. Although concrete data on TAVI-related new-onset CRT device implantations are still lacking, a number of recent studies have revealed that a significant number of patients with aortic valve stenosis developed persistent left bundle branch block (LBBB) after TAVI due to implantation-related damages of the cardiac conduction system (68, 69), making those patients potential candidates for a new-onset CRT device implantation. Moreover, the indications for TAVI (aortic valve stenosis) and CRT share similar risk factor such as, e.g., age and LV ejection fraction (70, 71). No data exist on whether TAVI or CRT candidates might have coronary vein (diameter, tributary topography) or SEAT morphologies different from other patients. There are no indications that this could be the case.

## Conclusions

Knowledge on SEAT thickness and allocation is hitherto scarce, although SEAT is a relevant electrophysiological factor in CRT due to its insulating properties, possibly interfering with both effective sensing and pacing. Besides CRT responsiveness of different areas of the LV myocardium and localization of the latest activated region, consideration of the individual SEAT conditions during electrode positioning might help to decrease undersensing and improve battery longevity. Our measurements and those of previous studies revealed a generally greater average SEAT thickness for the VIA compared to VVSP and VMS. This finding suggests that signal/noise ratios might be superior in VVSP and VMS, thus strengthening the preference for the lateral and posterolateral LV regions as optimal pacing regions for most CRT; the majority of recent clinical studies on CRT lead position indicate this. Leadless CRT systems could avoid problems with vein morphology and SEAT insulation but have their own limitations.

## Data Availability Statement

The raw data supporting the conclusions of this article will be made available by the authors, without undue reservation.

## Ethics Statement

The studies involving human participants were reviewed and approved by Rostock University Medical Center ethics committee (A 2019-0014). Written informed consent for participation was not required for this study in accordance with the national legislation and the institutional requirements.

## Author Contributions

JK and FM designed and planned the project. JK analyzed the measurement data and designed concepts for figures. JK, FM, AW, M-AW, JO, and FS interpreted and discussed the data. JK and FS wrote the manuscript. All authors have read and approved the article. All authors contributed to the article and approved the submitted version.

## Conflict of Interest

The authors declare that the research was conducted in the absence of any commercial or financial relationships that could be construed as a potential conflict of interest.

## Supplementary Material

Supplementary Figure 1 Direct comparison of venous diameters along the distance of the individual vein segments of the different studied single coronary vein samples. CS, coronary sinus; VCM, great cardiac vein; VIA, anterior interventricular vein; VMS, left marginal vein; VVSP, left posterior ventricular vein.

Supplementary Figure 2 Direct comparison of the subvenous epicardial adipose tissue (SEAT) thickness along the distance of the individual vein segments of the different studied single coronary vein samples. CS, coronary sinus; VCM, great cardiac vein; VIA, anterior interventricular vein; VMS, left marginal vein; VVSP, left posterior ventricular vein.
