# Supplementary material for: Morphometric Characterization of Human Coronary Veins and Subvenous Epicardial Adipose Tissue—Implications for Cardiac Resynchronization Therapy Leads
Source: Front Cardiovasc Med. 2020 Dec 8;7:611160. doi: 10.3389/fcvm.2020.611160 (PMC7793918; doi:10.3389/fcvm.2020.611160)
Supplement: Supplementary file 1 [file Table_1.DOCX]

**Supplementary Table 1.** Studied hearts from corpses (body donors) freshly taken and fixed for examination by mean of frozen section technique (FST) and micro computed tomography (mCT). EtOH = ethanol, n.a. = not available, PBS = phosphate buffered saline

| **corpse #** | **age [years]** | **sex** | **body length [cm]** | **post fixation** | **imaging** |
| --- | --- | --- | --- | --- | --- |
| 02/019 | 80 | f | 153 | PBS | FST |
| 83/018 | 81 | m | 175 | PBS | FST |
| 79/018 | 96 | f | 158 | PBS | FST |
| 68/018 | 94 | f | 162 | PBS | FST |
| 19/017 | 79 | m | 180 | EtOH | mCT |
| 16/018 | 97 | f | 142 | EtOH | mCT |
| 38/016 | 82 | m | n.a. | EtOH | mCT |
| 28/018 | 59 | f | 163 | EtOH | mCT |
| 42/018 | 91 | f | 148 | EtOH | mCT |
| 60/018 | 77 | m | 170 | EtOH | mCT |
| 12/016 | 91 | f | 160 | EtOH | mCT |
| 22/017 | 82 | f | n.a. | EtOH | mCT |
| 36/016 | 88 | m | n.a. | EtOH | mCT |
| 51/017 | 80 | m | 180 | EtOH | mCT |
