# Supplementary material for: Morphometric Characterization of Human Coronary Veins and Subvenous Epicardial Adipose Tissue—Implications for Cardiac Resynchronization Therapy Leads
Source: Front Cardiovasc Med. 2020 Dec 8;7:611160. doi: 10.3389/fcvm.2020.611160 (PMC7793918; doi:10.3389/fcvm.2020.611160)
Supplement: Supplementary file 2 [file Table_2.DOCX]

Supplementary Table 2. Overview of the venous diameters (VD) of coronary sinus (CS)/great cardiac vein (VCM), left posterior ventricular vein (VVSP), left marginal vein (VMS), and anterior interventricular vein (VIA). Values given for each position (pos) are in mm. Sample size (n) indicates number of measured segments for each position. * - measurement positions start distal to posterior interventricular vein.

|  | **CS/VCM VD*** | | | | | **VVSP VD** | | | | | **VMS VD** | | | | | **VIA VD** | | | | |
| --- | --- | --- | --- | --- | --- | --- | --- | --- | --- | --- | --- | --- | --- | --- | --- | --- | --- | --- | --- | --- |
| **pos** | **max** | **min** | **mean** | **SD** | **n** | **max** | **min** | **mean** | **SD** | **n** | **max** | **min** | **mean** | **SD** | **n** | **max** | **min** | **mean** | **SD** | **n** |
| **5** | 24.0 | 5.1 | 13.8 | 5.2 | 13 | 6.6 | 1.6 | 4.0 | 1.4 | 19 | 5.5 | 0.6 | 3.2 | 1.5 | 15 | 6.5 | 1.4 | 3.9 | 1.3 | 17 |
| **10** | 16.9 | 7.0 | 12.1 | 3.3 | 13 | 5.5 | 1.3 | 3.6 | 1.1 | 19 | 4.5 | 0.6 | 2.9 | 1.3 | 14 | 6.0 | 1.4 | 3.9 | 1.2 | 17 |
| **15** | 19.3 | 6.0 | 10.9 | 3.7 | 14 | 5.0 | 1.0 | 3.1 | 0.9 | 18 | 4.5 | 1.4 | 2.8 | 1.0 | 13 | 5.8 | 1.1 | 3.6 | 1.3 | 17 |
| **20** | 14.9 | 4.4 | 9.8 | 3.0 | 14 | 4.9 | 0.8 | 3.1 | 1.0 | 17 | 4.0 | 0.7 | 2.5 | 1.0 | 12 | 5.5 | 1.7 | 3.5 | 1.1 | 16 |
| **25** | 12.9 | 5.5 | 9.3 | 2.3 | 15 | 4.6 | 0.6 | 3.0 | 1.0 | 14 | 4.0 | 0.5 | 2.4 | 1.0 | 13 | 5.2 | 1.2 | 3.6 | 1.1 | 15 |
| **30** | 13.0 | 5.9 | 9.3 | 2.2 | 15 | 4.1 | 0.7 | 2.7 | 0.8 | 14 | 4.0 | 0.5 | 2.3 | 0.9 | 13 | 5.2 | 1.2 | 3.4 | 1.1 | 15 |
| **35** | 12.6 | 5.8 | 8.6 | 2.1 | 15 | 3.7 | 0.7 | 2.4 | 0.9 | 13 | 3.5 | 1.3 | 2.3 | 0.6 | 12 | 4.7 | 2.2 | 3.2 | 0.7 | 13 |
| **40** | 13.3 | 5.8 | 8.3 | 2.2 | 15 | 3.8 | 1.6 | 2.4 | 0.7 | 12 | 3.5 | 1.3 | 2.1 | 0.7 | 11 | 4.8 | 2.2 | 2.9 | 0.7 | 13 |
| **45** | 13.1 | 5.2 | 7.8 | 2.2 | 15 | 3.2 | 1.4 | 2.4 | 0.5 | 10 | 3.3 | 1.5 | 2.3 | 0.6 | 6 | 4.6 | 0.8 | 2.7 | 1.0 | 12 |
| **50** | 9.7 | 5.2 | 7.5 | 1.4 | 15 | 3.2 | 1.4 | 2.4 | 0.6 | 10 | 3.3 | 1.5 | 2.3 | 0.7 | 5 | 2.9 | 0.8 | 2.4 | 0.6 | 10 |
| **55** | 9.4 | 4.9 | 7.1 | 1.3 | 15 | 3.5 | 1.8 | 2.5 | 0.6 | 7 | 2.4 | 1.9 | 2.1 | 0.2 | 5 | 3.3 | 1.0 | 2.2 | 0.8 | 11 |
| **60** | 9.5 | 4.3 | 6.5 | 1.4 | 15 | 3.3 | 1.2 | 2.5 | 0.7 | 7 | 2.4 | 1.7 | 2.1 | 0.3 | 5 | 3.5 | 1.0 | 2.1 | 0.9 | 11 |
| **65** | 10.0 | 3.6 | 6.3 | 1.9 | 15 | 3.3 | 1.9 | 2.4 | 0.5 | 6 | 1.9 | 1.0 | 1.5 | 0.5 | 4 | 2.9 | 0.6 | 2.0 | 0.9 | 7 |
| **70** | 10.4 | 3.6 | 5.9 | 1.9 | 15 | 3.1 | 1.9 | 2.5 | 0.6 | 6 | 1.9 | 1.0 | 1.5 | 0.5 | 3 | 3.4 | 0.6 | 2.4 | 1.0 | 5 |
| **75** | 8.4 | 3.6 | 5.7 | 1.4 | 15 | 3.1 | 1.1 | 2.0 | 0.8 | 6 | 2.2 | 2.2 | 2.2 | - | 1 | 3.7 | 0.6 | 2.2 | 1.5 | 3 |
| **80** | 7.7 | 3.6 | 5.3 | 1.3 | 15 | 3.0 | 1.1 | 1.9 | 0.7 | 6 | 2.2 | 2.2 | 2.2 | - | 1 | 2.9 | 0.6 | 2.0 | 1.2 | 3 |
| **85** | 7.2 | 3.8 | 5.2 | 1.2 | 11 | 3.3 | 1.2 | 1.7 | 0.8 | 6 |  |  |  |  |  | 2.9 | 2.9 | 2.9 | - | 1 |
| **90** | 7.7 | 4.1 | 5.4 | 1.0 | 11 | 2.8 | 1.2 | 1.6 | 0.7 | 5 |  |  |  |  |  | 3.8 | 3.8 | 3.8 | - | 1 |
| **95** | 7.0 | 3.9 | 5.3 | 1.0 | 10 | 2.0 | 0.9 | 1.4 | 0.8 | 2 |  |  |  |  |  | 3.2 | 3.2 | 3.2 | - | 1 |
| **100** | 7.0 | 2.9 | 5.3 | 1.4 | 8 | 0.9 | 0.9 | 0.9 | - | 1 |  |  |  |  |  | 3.0 | 3.0 | 3.0 | - | 1 |
| **105** | 7.0 | 4.1 | 5.0 | 1.1 | 8 |  |  |  |  |  |  |  |  |  |  | 3.2 | 3.2 | 3.2 | - | 1 |
| **110** | 6.0 | 2.5 | 4.3 | 1.1 | 8 |  |  |  |  |  |  |  |  |  |  | 2.7 | 2.7 | 2.7 | - | 1 |
| **115** | 6.1 | 3.8 | 5.0 | 1.0 | 6 |  |  |  |  |  |  |  |  |  |  | 2.7 | 2.7 | 2.7 | - | 1 |
| **120** | 5.2 | 4.3 | 4.8 | 0.4 | 5 |  |  |  |  |  |  |  |  |  |  | 2.6 | 2.6 | 2.6 | - | 1 |
| **125** | 4.8 | 4.2 | 4.4 | 0.4 | 4 |  |  |  |  |  |  |  |  |  |  | 2.9 | 2.7 | 2.8 | - | 2 |
| **130** | 4.8 | 3.8 | 4.3 | 0.7 | 3 |  |  |  |  |  |  |  |  |  |  | 3.0 | 2.9 | 3.0 | - | 2 |
| **135** | 4.5 | 4.0 | 4.2 | 0.4 | 3 |  |  |  |  |  |  |  |  |  |  |  |  |  |  |  |
| **140** | 4.0 | 4.0 | 4.0 | - | 2 |  |  |  |  |  |  |  |  |  |  |  |  |  |  |  |
