# Supplementary material for: Morphometric Characterization of Human Coronary Veins and Subvenous Epicardial Adipose Tissue—Implications for Cardiac Resynchronization Therapy Leads
Source: Front Cardiovasc Med. 2020 Dec 8;7:611160. doi: 10.3389/fcvm.2020.611160 (PMC7793918; doi:10.3389/fcvm.2020.611160)
Supplement: Supplementary file 3 [file Table_3.DOCX]

Supplementary Table 3. Overview of the subvenous epicardial adipose tissue (SEAT) thickness of coronary sinus (CS)/great cardiac vein (VCM), left posterior ventricular vein (VVSP), left marginal vein (VMS), and anterior interventricular vein (VIA). Values given for each position (pos) are in mm. Sample size (n) indicates number of measured segments for each position. * - measurement positions start distal to posterior interventricular vein.

|  | **CS/VCM SEAT*** | | | | | **VVSP SEAT** | | | | | **VMS SEAT** | | | | | **VIA SEAT** | | | | |
| --- | --- | --- | --- | --- | --- | --- | --- | --- | --- | --- | --- | --- | --- | --- | --- | --- | --- | --- | --- | --- |
| **pos** | **max** | **min** | **mean** | **SD** | **n** | **max** | **min** | **mean** | **SD** | **n** | **max** | **min** | **mean** | **SD** | **n** | **max** | **min** | **mean** | **SD** | **n** |
| **5** | 9.6 | 2.5 | 5.3 | 2.3 | 10 | 11.1 | 1.0 | 4.3 | 2.7 | 17 | 7.3 | 1.2 | 3.6 | 2.2 | 14 | 9.9 | 1.3 | 4.4 | 2.2 | 16 |
| **10** | 7.4 | 2.8 | 4.6 | 1.6 | 10 | 8.4 | 0.5 | 3.2 | 2.2 | 17 | 8.5 | 0.8 | 3.7 | 2.7 | 13 | 9.6 | 0.3 | 3.6 | 2.9 | 17 |
| **15** | 6.8 | 1.7 | 4.0 | 1.8 | 12 | 6.0 | 0.5 | 2.1 | 1.6 | 18 | 7.9 | 0.4 | 2.8 | 2.2 | 13 | 10.6 | 0.4 | 4.6 | 3.2 | 15 |
| **20** | 9.3 | 1.7 | 4.7 | 2.6 | 12 | 4.5 | 0.5 | 1.8 | 1.3 | 17 | 3.6 | 0.3 | 1.7 | 1.1 | 12 | 10.4 | 0.5 | 4.5 | 3.2 | 15 |
| **25** | 9.5 | 1.5 | 4.7 | 2.5 | 14 | 4.6 | 0.3 | 1.2 | 1.1 | 15 | 4.5 | 0.1 | 1.7 | 1.3 | 12 | 12.4 | 1.3 | 4.7 | 3.1 | 15 |
| **30** | 9.6 | 1.5 | 4.7 | 2.5 | 14 | 4.1 | 0.3 | 1.0 | 1.1 | 12 | 4.4 | 0.1 | 1.8 | 1.3 | 12 | 12.2 | 0.4 | 5.0 | 2.9 | 15 |
| **35** | 10.2 | 0.1 | 5.2 | 3.2 | 13 | 3.1 | 0.2 | 1.0 | 0.8 | 13 | 2.4 | 0.1 | 1.1 | 0.7 | 12 | 9.7 | 1.5 | 4.8 | 2.9 | 13 |
| **40** | 12.6 | 0.1 | 5.6 | 4.0 | 13 | 3.1 | 0.2 | 1.4 | 1.0 | 12 | 2.3 | 0.1 | 1.1 | 0.6 | 11 | 9.7 | 1.0 | 4.7 | 2.7 | 12 |
| **45** | 15.3 | 0.1 | 6.1 | 4.2 | 14 | 2.0 | 0.2 | 0.9 | 0.6 | 10 | 2.1 | 0.3 | 1.2 | 0.7 | 6 | 8.0 | 0.9 | 3.4 | 2.1 | 11 |
| **50** | 14.8 | 0.1 | 6.3 | 4.2 | 14 | 2.0 | 0.2 | 0.8 | 0.6 | 9 | 2.8 | 0.4 | 1.7 | 1.1 | 5 | 7.5 | 0.8 | 3.5 | 2.1 | 9 |
| **55** | 11.3 | 0.2 | 6.5 | 3.8 | 14 | 3.4 | 0.5 | 1.2 | 1.1 | 7 | 4.4 | 0.2 | 2.1 | 1.6 | 5 | 6.8 | 1.2 | 4.2 | 2.1 | 9 |
| **60** | 11.6 | 0.2 | 5.9 | 3.7 | 14 | 4.1 | 0.5 | 1.7 | 1.3 | 7 | 2.8 | 0.2 | 1.6 | 1.1 | 5 | 6.7 | 0.6 | 3.6 | 2.1 | 10 |
| **65** | 12.0 | 1.8 | 5.9 | 3.3 | 14 | 3.1 | 0.5 | 1.5 | 1.0 | 7 | 3.9 | 0.1 | 1.9 | 1.6 | 4 | 5.7 | 1.3 | 3.3 | 1.7 | 7 |
| **70** | 10.3 | 2.9 | 6.0 | 2.4 | 14 | 3.6 | 0.9 | 2.2 | 1.1 | 6 | 3.6 | 0.1 | 1.8 | 1.8 | 3 | 5.3 | 2.2 | 3.4 | 1.2 | 5 |
| **75** | 10.8 | 2.6 | 6.1 | 2.6 | 13 | 4.3 | 0.3 | 2.3 | 1.4 | 6 |  |  |  |  |  | 4.1 | 0.6 | 2.5 | 1.7 | 3 |
| **80** | 9.0 | 2.5 | 5.4 | 2.2 | 13 | 4.5 | 0.3 | 2.3 | 1.5 | 6 |  |  |  |  |  | 4.5 | 0.6 | 2.2 | 2.1 | 3 |
| **85** | 9.1 | 2.8 | 5.8 | 1.7 | 10 | 4.1 | 0.1 | 2.0 | 1.6 | 6 |  |  |  |  |  | 2.2 | 2.2 | 2.2 | - | 1 |
| **90** | 7.8 | 3.1 | 5.7 | 1.5 | 10 | 4.1 | 0.1 | 2.3 | 1.7 | 6 |  |  |  |  |  | 2.5 | 2.5 | 2.5 | - | 1 |
| **95** | 7.8 | 2.1 | 5.3 | 1.9 | 9 | 3.0 | 1.1 | 2.0 | 1.3 | 2 |  |  |  |  |  | 2.2 | 2.2 | 2.2 | - | 1 |
| **100** | 6.0 | 0.7 | 3.8 | 1.9 | 7 | 1.1 | 1.1 | 1.1 | - | 1 |  |  |  |  |  | 2.4 | 2.4 | 2.4 | - | 1 |
| **105** | 7.0 | 0.9 | 3.7 | 2.4 | 7 |  |  |  |  |  |  |  |  |  |  | 3.0 | 3.0 | 3.0 | - | 1 |
| **110** | 7.4 | 0.5 | 3.3 | 2.4 | 7 |  |  |  |  |  |  |  |  |  |  | 3.5 | 3.5 | 3.5 | - | 1 |
| **115** | 8.8 | 1.2 | 4.2 | 2.9 | 5 |  |  |  |  |  |  |  |  |  |  | 4.1 | 4.1 | 4.1 | - | 1 |
| **120** | 2.8 | 1.8 | 2.2 | 0.5 | 4 |  |  |  |  |  |  |  |  |  |  | 4.5 | 4.5 | 4.5 | - | 1 |
| **125** | 2.7 | 0.7 | 1.9 | 1.0 | 3 |  |  |  |  |  |  |  |  |  |  | 2.4 | 2.4 | 2.4 | - | 1 |
| **130** | 3.0 | 0.8 | 1.9 | 1.6 | 2 |  |  |  |  |  |  |  |  |  |  | 1.8 | 1.8 | 1.8 | - | 1 |
| **135** | 3.3 | 1.1 | 2.2 | 1.6 | 2 |  |  |  |  |  |  |  |  |  |  |  |  |  |  |  |
| **140** | 1.5 | 1.5 | 1.5 | - | 1 |  |  |  |  |  |  |  |  |  |  |  |  |  |  |  |
