# Supplementary figures and images for: Morphometric Characterization of Human Coronary Veins and Subvenous Epicardial Adipose Tissue—Implications for Cardiac Resynchronization Therapy Leads
Source: Front Cardiovasc Med. 2020 Dec 8;7:611160. doi: 10.3389/fcvm.2020.611160 (PMC7793918; doi:10.3389/fcvm.2020.611160)

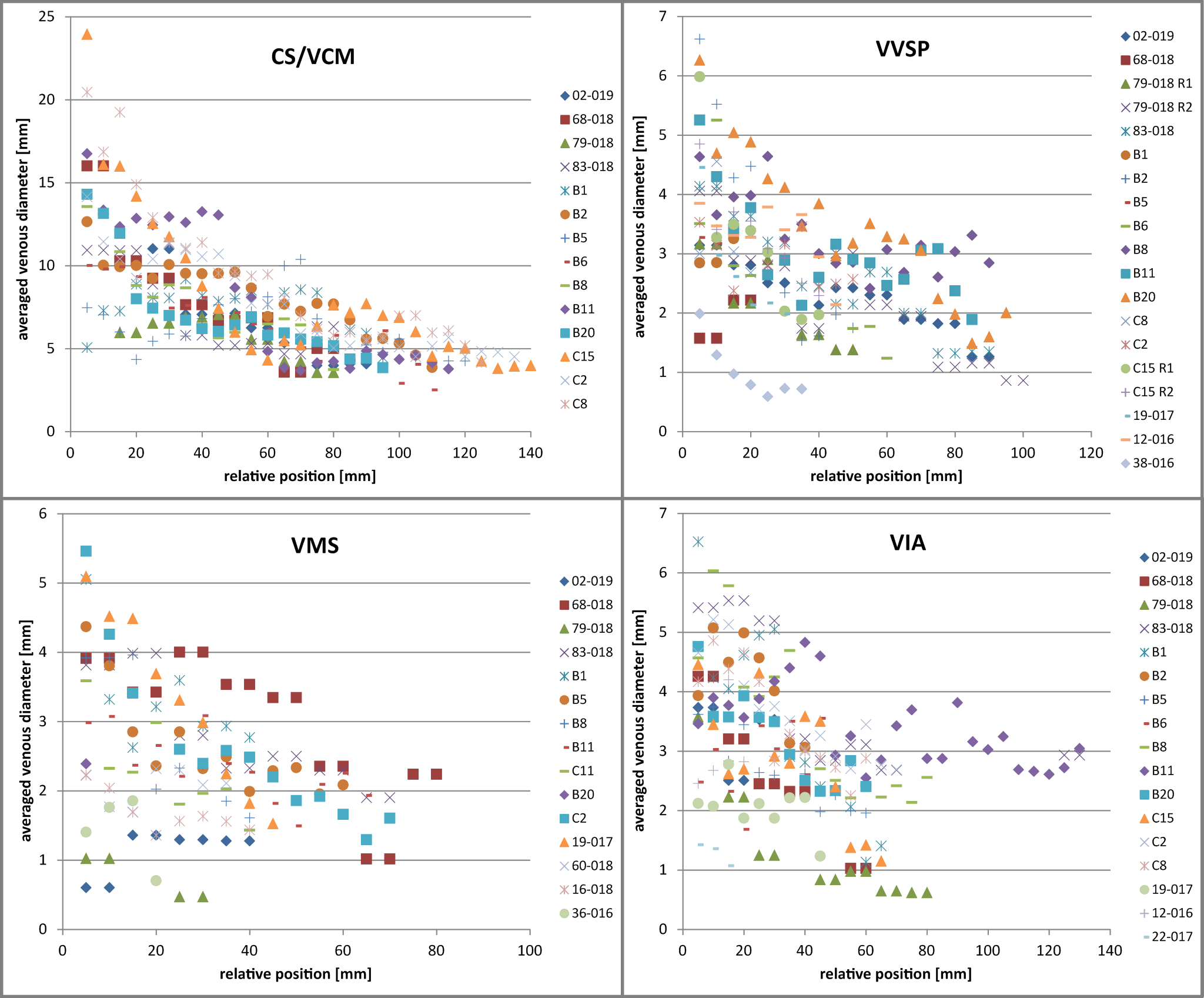

Supplement: Supplementary Figure 1 — Direct comparison of venous diameters along the distance of the individual vein segments of the different studied single coronary vein samples. CS, coronary sinus; VCM, great cardiac vein; VIA, anterior interventricular vein; VMS, left marginal vein; VVSP, left posterior ventricular vein. [file Image_1.TIF]

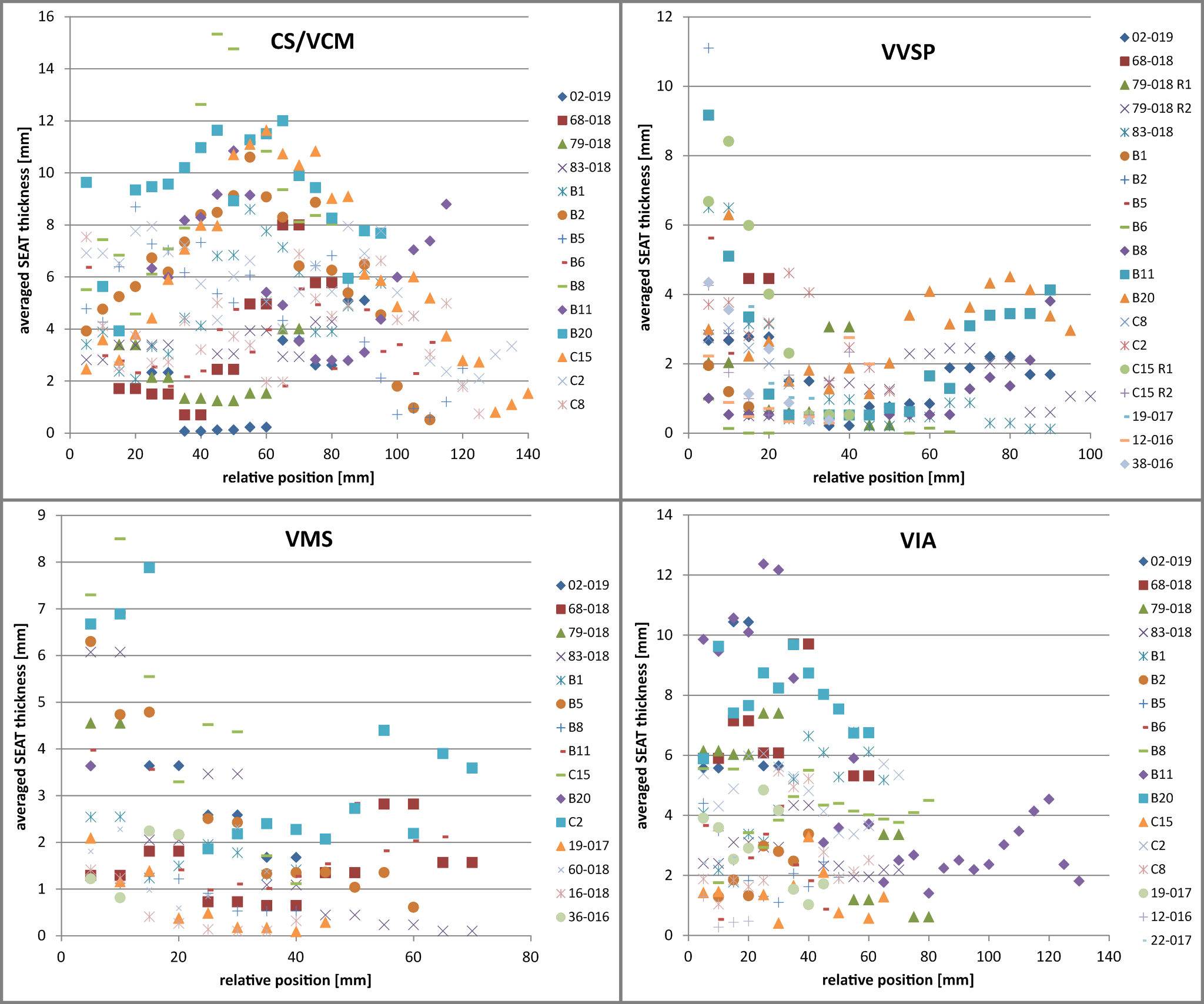

Supplement: Supplementary Figure 2 — Direct comparison of the subvenous epicardial adipose tissue (SEAT) thickness along the distance of the individual vein segments of the different studied single coronary vein samples. CS, coronary sinus; VCM, great cardiac vein; VIA, anterior interventricular vein; VMS, left marginal vein; VVSP, left posterior ventricular vein. [file Image_2.TIF]
